# The effects of different frequency treadmill exercise on lipoxin A_4_ and articular cartilage degeneration in an experimental model of monosodium iodoacetate-induced osteoarthritis in rats

**DOI:** 10.1371/journal.pone.0179162

**Published:** 2017-06-08

**Authors:** Yue Yang, Yang Wang, Yawei Kong, Xiaoning Zhang, Lunhao Bai

**Affiliations:** 1 Department of Orthopedic Surgery, Shengjing Hospital, China Medical University, ShenYang, Liaoning, China; 2 Department of Ultrasound, Shengjing Hospital, China Medical University, ShenYang, Liaoning, China; 3 International Patient Center, Brigham and Women’s Hospital, Harvard Medical School, Boston, Massachusetts, United States of America; University of Umeå, SWEDEN

## Abstract

The aim was to investigate the effects of different frequencies treadmill exercise with total exercise time being constancy on articular cartilage, lipoxin A_4_ (LXA_4_) and the NF-κB pathway in rat model of monosodium iodoacetate-induced osteoarthritis (OA). Fifty male Sprague-Dawley rats were randomly divided into five groups (n = 10): controls (CG), knee OA model (OAG), OA + treadmill exercise once daily (OAE1), OA + treadmill exercise twice daily, rest interval between exercise>4h (OAE2) and OA + treadmill exercise three times daily, rest interval between exercise>4h (OAE3). Rats were evaluated after completing the treadmill exercise program (speed, 18 m/min; total exercise time 60 min/day; 5 days/week for 8 weeks). Interleukin (IL)-1β, tumor necrosis factor (TNF)-α, and LXA_4_ in serum and intra-articular lavage fluid were measured by ELISA. Changes in articular cartilage were evaluated by histology, immunohistochemistry, western blotting and quantitative real-time-PCR. LXA_4_ in the serum and intra-articular lavage fluid increased in all OAE groups, and histological evaluation indicated that the OAE3 group had the best treatment response. The expression of COL2A1 and IκB-β in articular cartilage increased in all OAE groups *vs* the OAG group, whereas expression of IL-1β, TNF-α, matrix metalloproteinase (MMP)-13, and NF-κB p65 was reduced in all OAE groups compared with the OAG. Under the condition of 60 min treadmill exercise with moderate-intensity, to fulfill in three times would have better chondroprotective effects than to fulfill in two or one time on monosodium iodoacetate-induced OA in rats. And it may be worked through the anti-inflammatory activity of LXA_4_ and the NF-κB pathway.

## Introduction

With ongoing worldwide increases in both aging and the size of obese populations, health professionals need to prepare for corresponding increases in the need to treat patients with hip and knee osteoarthritis (OA) [1]. OA is a chronic joint disease, characterized by the degeneration of articular cartilage, subchondral bone porosis, and inflammation of synovial membranes, all of which influence the structure and function of joints [2].

Many previous studies have confirmed that the joint changes seen in OA can be induced by 8 weeks of treatment with monosodium iodoacetate (MIA) [3]. Intra-articular injection of MIA in animal models (such as rats) results in pathological changes closely resembled those seen in human OA [4–8].

Epidemiological studies indicate that inflammatory molecules, including cytokines and adipokines, have been shown to damage joints [9]. Increased inflammation of joints plays an important role in the pathology of OA through synovitis [10,11]. Therefore studying inflammatory molecules is an effective approach toward understanding the pathophysiology of OA. The role of adipokines, which are produced by adipose tissue and released into blood participating in low-grade inflammation, has been widely studied in recent years [12]. Lipoxin A_4_ (LXA_4_) is a potent stop signal of inflammation [13] and its therapeutic effect on OA has been confirmed by previous studies [14–16]. LXA_4_ can be formed by the action of 12-lipoxygenase in the capillaries [17] and the activation of the oxylipin pathway of the infrapatellar fat pad [18].

NF-κB signaling pathway plays a central role in regulation of inflammation and OA pathogenesis [19]. NF-κB p65 is thought to be a link between tensile loading and chondrocytic responses to pro-inflammatory cytokines, and activation of NF-κB p65 is a key event in NF-κB-mediated induction of IL-1β, TNF-α, and matrix metalloproteinase (MMP) gene expression [20].

OA can induce pain, stiffness, and even disability of joints [21]. Adequate exercise has been shown to be beneficial in people with OA by relieving pain and increasing mobility [22]. Although physical exercise is one of the most common nonpharmacological OA therapies, the duration and type of exercise programs varied widely [23]. Speed, duration and frequency are three main variables related to treadmill exercise. Although moderate treadmill exercises are known to maintain the integrity of cartilage, the relationship between frequency of treadmill exercise and OA progression are still unclear [24–32].

Our preliminary experiment found that LXA_4_ was rapidly elevated in 2 hours and rapidly reduced to normal level at 4 hours after treadmill exercise. And the variation of LXA_4_ was similar after 20min, 30min and 60min treadmill exercise (S1 Fig). Therefore, the purpose of this study was to investigate the effect of different frequencies with total exercise time being constancy on cartilage, LXA_4_ and NF-κB pathway in a rat model of OA induced by MIA. The results provide new insight on mechanism about how the exercise exert treatment role on OA.

## Materials and methods

### Experimental animals

Fifty male Sprague-Dawley (SD) rats (230 ± 10 g, 8 weeks of age, and specific-pathogen-free) were obtained from HFK Bioscience Co. Ltd. (Beijing, China). Maintenance and care of the experimental rats followed the guidelines of the Ethics Committee of Shengjing Hospital of China Medical University, and this study was approved by this Ethics Committee. Rats were kept in individual plastic cages on sawdust bedding, a 12:12 h light:dark cycle with the lights on from 6:00 a.m. to 6:00 p.m., a controlled temperature of 22 ± 2°C, and 70% humidity. They had free access to a planned diet. Body weight was recorded at regular intervals. They were adapted to laboratory conditions for 1 week prior to the experimental procedures. And then, all rats were habituated to the treadmill exercise for 1 week at a speed of 10 m/min for 10 min/day to reduce stress. All rats adapted to the treadmill exercise.

### OA model and treadmill running protocols

**Fig 1:**
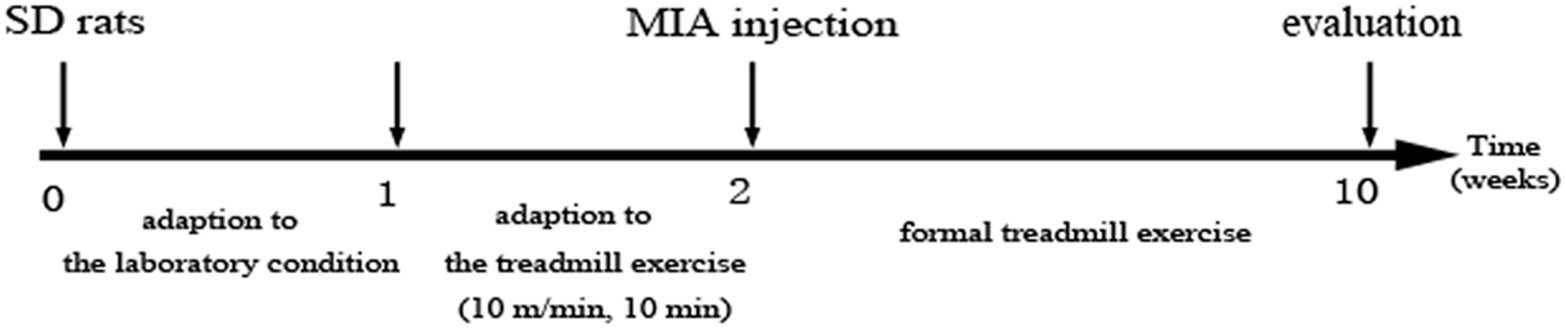
The design of the treatment schedule and intervals of various parameters.

After the adaptive treadmill exercise, the rats were numbered from 1 to 50 and randomly grouped by excel. And then, fifty SD rats were randomly divided into five groups (n = 10): a control group (CG); knee OA model group (OAG); OA + treadmill exercise once daily (OAE1); OA + treadmill exercise twice daily (OAE2); and OA + treadmill exercise three times daily (OAE3). And then, all rats were anesthetized with 1.5% pentobarbital sodium (0.2 ml/100 g, intraperitoneal injection). Knee joint inflammation was induced by intra-articular injection of MIA (1 mg per cavity in 50 μL sterile saline) by micro syringe through the suprapatellar ligament and into the bilateral knee joint cavity. The CG rats received an intra-articular injection of 50 μL sterile saline. All rats were kept sedentary, but the OAE groups began their exercise programs 24 h after injection (Fig 1). As to exercise speed for rats, moderate running protocol was 18 m/min according to the 70% maximal O_2_ [33]. Total 60min/day, and 5 days/week in all OAE groups. OAE1 animals exercised 60 min once daily, OAE2 exercised 30 min twice daily, and OAE3 exercised 20 min three times daily (Table 1). And our preliminary experiment found that LXA_4_ was rapidly elevated in 2 hours and rapidly reduced to basal level at 4 hours after treadmill exercise (S1 Fig). We designed the intervals between two treadmill exercises > 4 hours.

**Table 1:** Training protocolss for OA rat model study groups.

Group	Speed (m/min)	Duration (min)	Frequency (times/day)
CG	0	0	0
OAG	0	0	0
OAE1	18	60	1
OAE2	18	30	2
OAE3	18	20	3

### Sampling and tissue preparation

After 8 weeks of exercise, blood samples were obtained immediately after the animals were anesthetized, and centrifuged at 3000 *g* for 10 min to obtain serum. The left knee joints of all rats were dissected and fixed in 10% formaldehyde solution. Intra-articular lavage fluid was obtained from the synovial cavity of the right knee by injection and recovery of 0.3 ml of phosphate-buffered saline (PBS) for 3 times using a 1 ml syringe. Articular cartilage specimens were removed from the weight-bearing area of the condyles of the right femur and tibia using a scalpel. All tissues were stored at −80°C immediately.

### Histology

Left knee joint tissue samples were stored in 10% formaldehyde solution for 7 days. Then they were washed in water for 5 h and transferred to 20% EDTA solution (Jianglai Reagent Co., Ltd, Shanghai, China) to decalcify for 21 days and the solution was changed every 3 days. Decalcified samples were dehydrated in an ethanol series and embedded in paraffin. Serial 5 μm sagittal sections were cut from the tibiofemoral joints for histological examination. The sections were stained with hematoxylin and eosin (HE) and toluidine blue to observe the cartilage. Injury of the articular cartilage in femur and tibia was assessed by the Modified Mankin score [34] on a scale of 0–14 points and the Osteoarthritis Research Society International (OARSI) score [35,36] on a scale of 0–24 points. As both the tibial and femoral cartilages were evaluated, the maximum Mankin score was 28 and the maximum OARSI score was 48. Two experienced observers (Yue Yang and Xiaoning Zhang) performed the scoring in a blinded manner.

### Enzyme-linked immunosorbent assay (ELISA) of intra- articular lavage fluid and serum

TNF-α, IL-1β, and LXA_4_ levels in the knee intra-articular lavage fluid and in serum were determined using ELISA kits (TongWei, Shanghai, China) following the manufacturer’s instructions. And then, the content of protein in intra-articular lavage fluid were measured to ensure that the ratio of dilution were equal.

### Immunohistochemistry

In addition to histomorphological evaluation, serial sections were stained for COL2A1, NF-κB p65, MMP-13 and TIMP-1. After deparaffinization and rehydration of the tissue sections, the proteins were immunostained using a two-step method following the kit manufacturer’s instructions. The sections were incubated with rabbit polyclonal anti-COL2A1 antibody (sc-28887, 1:50; Santa Cruz), mouse monoclonal anti-NF-κB p65 antibody (sc-8008, 1:50; Santa Cruz), goat polyclonal anti-MMP-13 antibody (sc-31813, 1:50; Santa Cruz), or rabbit polyclonal anti-TIMP-1 antibody (sc-5538, 1:50; Santa Cruz) overnight at 4°C. The slides were washed three times in PBS followed by a 20 min incubation at 37°C with an anti-mouse/rabbit IgG detection system (PV-9000, Zhongshan Goldenbridge Biotechnology Co., China), anti-goat IgG detection system (PV-9003, Zhongshan Goldenbridge Biotechnology Co., Beijing, China) and visualized with diaminobenzidine (DAB). Nuclei were counterstained with hematoxylin for 5 min. Negative control sections were prepared with the same protocol as above, but the primary antibody was replaced by PBS. The optical density of stained slides was measured using image analysis software (NikonH600L Microscope and image analysis system, Japan). COL2A1 was expressed by relative intensity. NF-κB (p65), MMP-13 and TIMP-1 were expressed by percentage of positive cells.

### Western blotting

Chondrocytes were washed twice in ice-cold PBS and sonicated in lysis buffer (P0013C, Beyotime, China). The cell lysates centrifuged at 14 000 *g* for 5 min at 4°C. The protein concentration of the cell supernatants was measured with a bicinchoninic acid (BCA) assay kit (P0010S, Beyotime, China). Equal amounts of protein (50 μg) were separated by SDS-PAGE and transferred to polyvinylidene difluoride membranes. After blocking with 1% bovine serum albumin (BSA) in Tris-buffered saline (TBS) with 0.1% Tween-20 (TBST) at room temperature for 1 h, the blots were incubated overnight at 4°C with primary antibodies: goat polyclonal anti-MMP-13 (sc-31813, 1:200; Santa Cruz), molecular weight 48kDa; rabbit polyclonal anti-TIMP-1 antibody (sc-5538, 1:200; Santa Cruz), molecular weight 23 kDa; mouse monoclonal anti-NF-κB p65 antibody (sc-8008, 1:200; Santa Cruz), molecular weight 65 kDa; rabbit polyclonal anti-IκB-β antibody (sc-945, 1:200; Santa Cruz), molecular weight 45 kDa; rabbit polyclonal anti-COL2A1 antibody (100089, 1:200; TongWei), molecular weight 190 kDa; and mouse monoclonal anti-β-actin (60008-1-lg, 1:2000, Proteintech Group), molecular weight 42 kDa. After washing three times with TBST, the membranes were incubated with IgG-horseradish peroxidase (HRP)-conjugated secondary antibodies (1:10000, Canlife) at room temperature for 1 h. After washing with TBST buffer, immunoreactivity was detected with enhanced chemiluminescence (ECL) and quantified using Quantity ONE (Bio-Rad, Hercules, CA, USA) software. β-actin was used as the internal control.

### Quantitative real-time polymerase chain reaction (qPCR)

Rats were anesthetized and killed by cervical dislocation. Knee joint cartilage samples were homogenized in Trizol reagent and total RNA was extracted. cDNA was reverse transcribed from 1 μg of total RNA using a PrimeScript RT reagent kit with gDNA Eraser (Takara Bio, Dalian, China) according to the manufacturer's instructions. qPCR was performed in an ABI Prism 7500 Fast Real-Time PCR System (Applied Biosystems, Wilmington, NC, USA) using SYBR Premix Ex Taq II (Tli RNaseH Plus; Takara Bio, Dalian, China). Expression levels were calculated by the 2^-ΔΔCT^ method [37] with β-actin as the reference expression gene using the primers shown in Table 2.

**Table 2:** Sequences of primers used for qPCR.

Target gene	Forward primer 5′-3′	Reverse primer 5′-3′
MMP-13	TGATGATGAAACCTGGACAAGCA	GAACGTCATCATCTGGGAGCA
TIMP-1	CATCTCTGGCCTCTGGCATC	CATAACGCTGGTATAAGGTGGTCTC
β-actin	GGAGATTACTGCCCTGGCTCCTA	GACTCATCGTACTCCTGCTTGCTG

### Statistical analysis

Data were expressed as means with 95% confidence intervals (CI) and analyzed using SPSS statistical software version 16 (SSPS, Inc., Chicago, IL, USA). Shapiro-Wilk's and Levene's test were applied to evaluate the normality and homogeneity of the results, respectively. For the variables that exhibited normal distribution, one-way ANOVA and post-hoc Tukey tests were used for the statistical analysis of significance. For the variables that exhibited non-normal distribution, Kruskal-Wallis test was used. *P*-values <0.05 were accepted as significant.

## Results

### Histological observations

**Fig 2:**
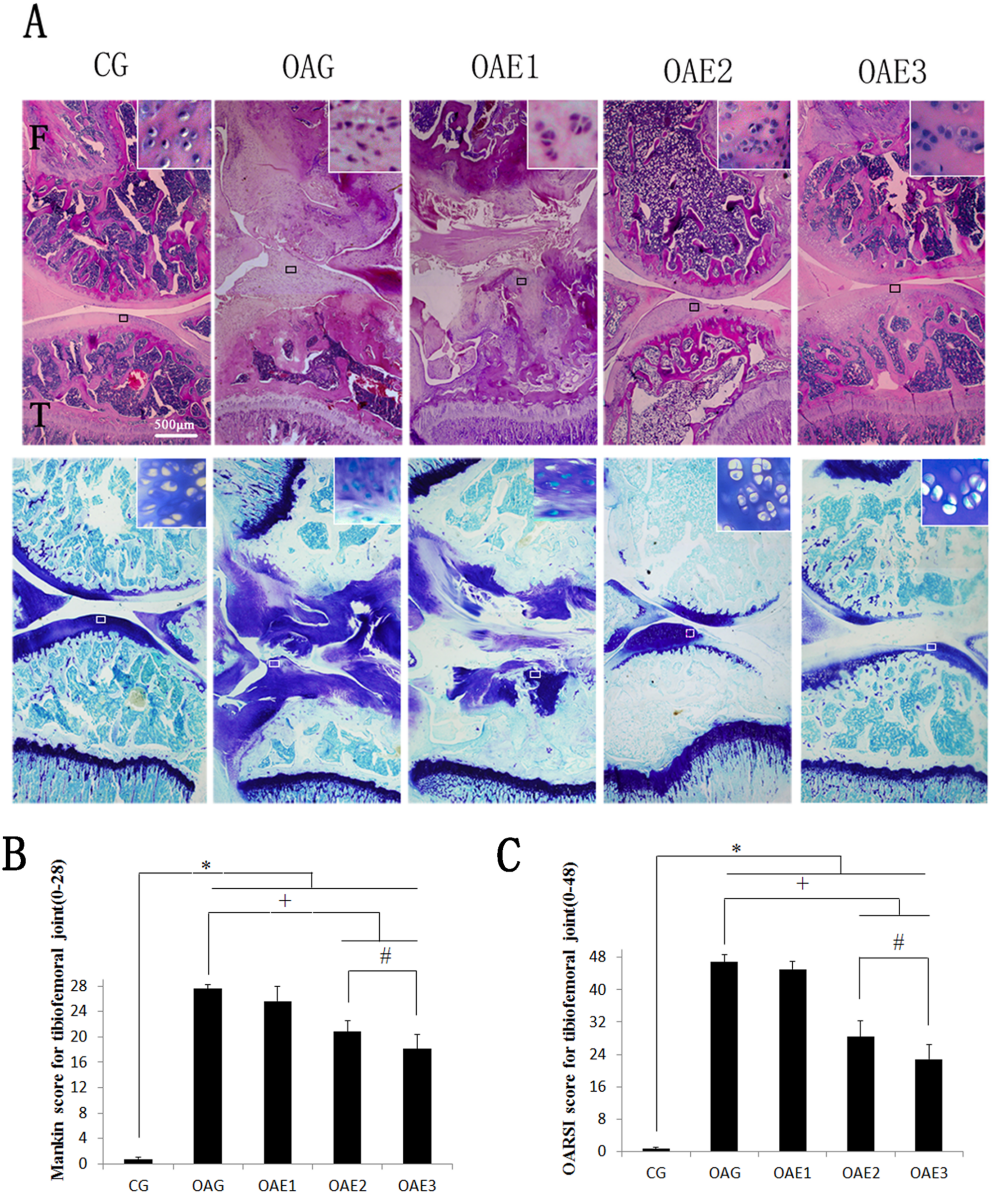
Histological evaluation of tibiofemoral joints. (A) Histological features of representative tibiofemoral joints sectioned in the sagittal plane and stained with HE and toluidine blue. Mankin and OARSI histological scores are shown for each image. F: femur, T: tibia. (B) Mankin score for tibiofemoral joints. Differences between CG and OAG (*P <0.001), OAG vs. OAE2 and OAE3 groups (^+^*P* <0.001) were significant, and between OAE2 and OAE3 (^#a^*P* = 0.006) were significant. (C) OARSI histological scores for cartilage of tibiofemoral joints. Differences between CG and OAG (*P <0.001), OAG vs. OAE2 and OAE3 (^+^*P* <0.001) were significant, and between OAE2 and OAE3 (^#b^*P* = 0.026) were significant. Kruskal-Wallis test, n = 10 rats in each group, means with 95% confidence interval.

Histological assessment (Mankin and OARSI score) demonstrated the damage of cartilage in OAG were serious compared to CG (Mankin score of tibiofemoral joints: CG = 0.9–95% CI 0.5–1.3; OAG = 26.6–95% CI 25.7–27.5; OARSI score of tibiofemoral joints: CG = 0.9–95% CI 0.3–1.5; OAG = 46.8–95% CI 44.9–48.7). OAE2 and OAE3 both had therapeutic effects on cartilage compared to OAG in tibiofemoral joints. But OAE3 obtained a better histological result than OAE1 and OAE2 in tibiofemoral joints (Mankin score of tibiofemoral joints: OAE1 = 25.4–95% CI 24.1–26.7; OAE2 = 20.0–95% CI 19.0–21.0; OAE3 = 17.8–95% CI 16.7–18.9; OARSI score of tibiofemoral joints: OAE1 = 45.6–95% CI 42.8–48.4; OAE2 = 33.2–95% CI 30.5–35.9; OAE3 = 27.6–95% CI 23.2–32.0) (Fig 2A, 2B and 2C).

### ELISA of serum and intra-articular lavage fluid

**Fig 3:**
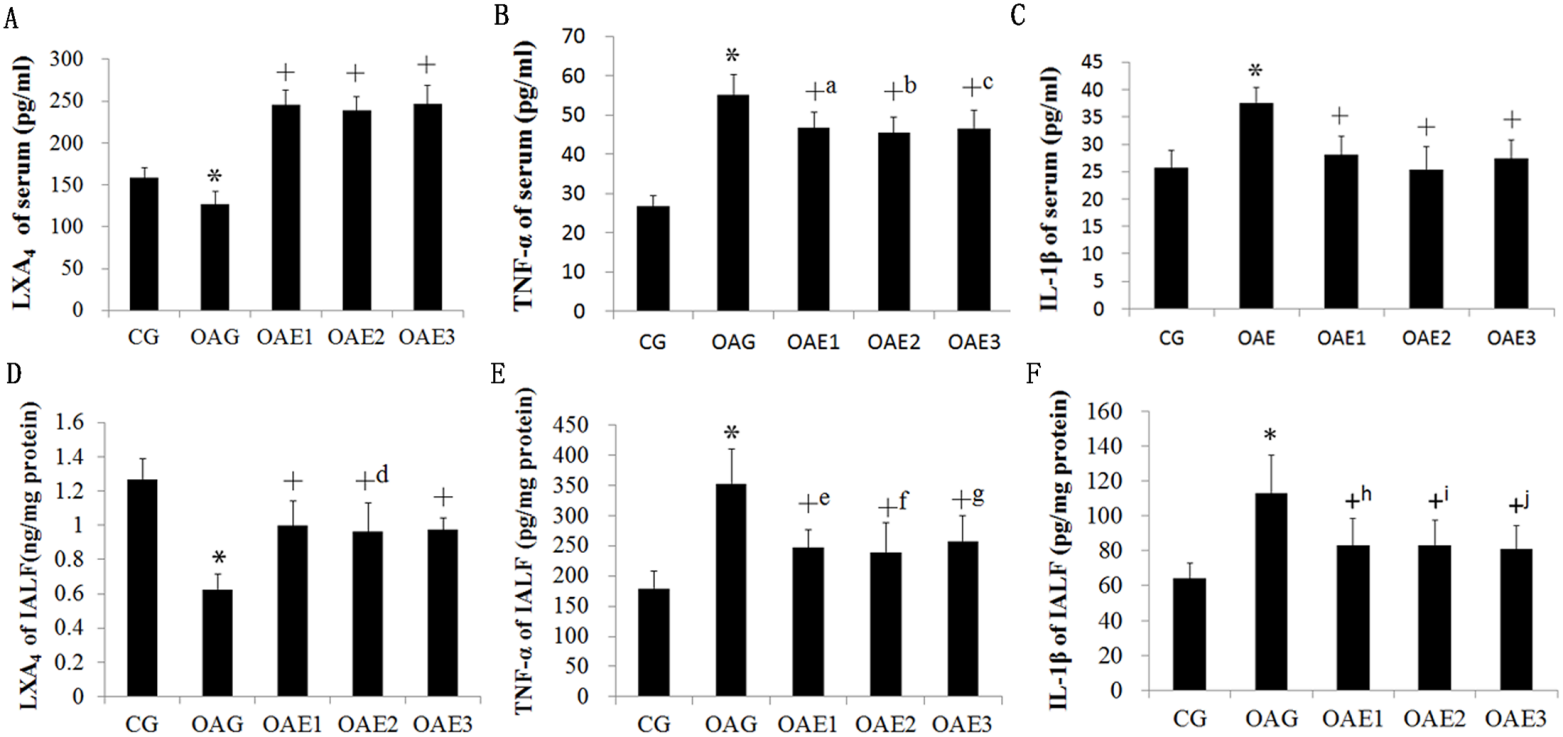
ELISA results in each group. Each treadmill frequency significantly alleviated the serum and articular inflammatory responses induced by MIA, including LXA_4_ (A,D), IL-1β (B,E), and TNF-α (C,F). Differences between CG and OAG were significant (*P <0.001), and differences between the OAG group and different frequency treadmill groups were significant (^+^*P*<0.001, ^+a^*P* = 0.010, ^+b^*P* = 0.002, ^+c^*P* = 0.003, ^+d^*P* = 0.001, ^+e^*P* = 0.002, ^+f^*P* = 0.001, ^+g^*P* = 0.007, ^+h^*P* = 0.024, ^+i^*P* = 0.027, ^+j^*P* = 0.013). One-way ANOVA, n = 10 rats for each group, means with 95% confidence interval.

As shown in Fig 3, serum LXA_4_ concentration was lower in in the OAG than in the CG and higher in all treadmill groups than in the OAG (LXA_4_ in serum: CG = 158.6–95% CI 146.4–170.8 pg/ml; OAG = 126.6–95% CI 111.0–142.2 pg/ml; OAE1 = 245.6–95% CI 227.7–263.5 pg/ml; OAE2 = 238.6–95% CI 221.3–255.8 pg/ml; OAE3 = 246.5–95% CI 224.6–268.4 pg/ml). (Fig 3A) LXA_4_ activity in the intra-articular lavage fluid was higher in the treadmill groups than in the OAG (LXA_4_ in intra-articular lavage fluid: CG = 1.27–95% CI 1.15–1.39 ng/mg protein; OA = 0.63–95% CI 0.54–0.71 ng/mg protein; OAE1 = 1.00–95% CI 0.85–1.14 ng/mg protein; OAE2 = 0.96–95% CI 0.79–1.13 ng/mg protein; OAE3 = 0.97–95% CI 0.90–1.05 ng/mg protein). (Fig 3D) Serum TNF-α and IL-1β concentrations were both higher in the OAG than in the CG; and each treadmill frequency reduced the elevation of serum TNF-α and IL-1β concentration compared with that seen in the OAG group. (Fig 3B and 3C) The changes of TNF-α and IL-1β concentration in intra-articular lavage fluid were similar to those observed in serum. (Fig 3E and 3F)

### Immunohistochemical analysis

**Fig 4:**
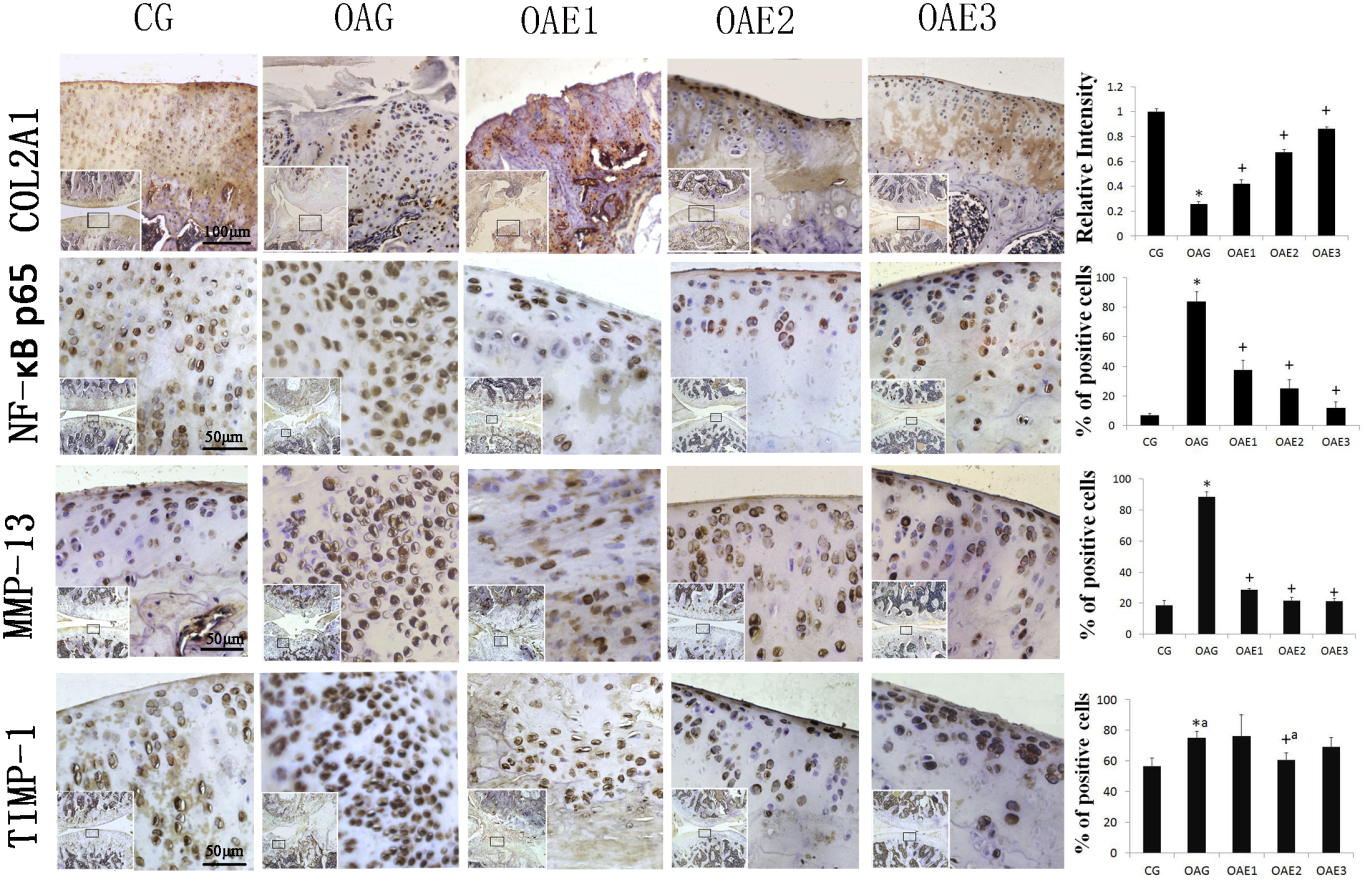
Immunohistochemical staining in each group. Increase in the frequency with same amount of treadmill exercise decreased the MIA-induced degradation of COL2A1 in articular cartilage and reduced the expression of MMP-13 and NF-κB p65. The micrographs showed the intensity of immunohistochemical staining of COL2A1, NF-κB p65, MMP-13, and TIMP-1 in the articular cartilage of each experimental group, and the figures showed the percentages of positively stained cells. Differences between CG and OAG were significant (*P <0.001, *^a^*P* = 0.001), and differences between OAG and the treadmill exercise groups were significant (^+^*P*<0.001, ^+a^*P* = 0.012). One-way ANOVA, n = 5 rats for each group, mean score with 95% confidence interval.

Immunohistochemical staining showed that increase in the frequency of treadmill exercise resulted in increased COL2A1 expression in articular cartilage compared with the OAG group. Rats in the OAE3 group also had higher COL2A1 expression than rats in the OAE1 and OAE2 groups (Fig 4).

The activation of NF-κB expression involves a shift from the cytoplasm into the nucleus. Compared with 84% in the OAG group, the percentage of NF-κB p65-positive cells in the articular cartilage was reduced to 37% in the OAE1, 24% in the OAE2, and 12% in the OAE3 group. The percentage of NF-κB p65-positive cells in the CG was 7%.

And the percentage of MMP-13-positive cells in the articular cartilage was reduced from 88% in the OAG group to 29% in the OAE1, 22% in the OAE2 and 21% in the OAE. The percentage of MMP-13-positive cells in the CG group was 19%. The percentage of TIMP-1-positive cells in articular cartilage was 75% in the OAG group, 76% in the OAE1, 61% in the OAE2, and 69% in the OAE3 group. The percentage of TIMP-1-positive cells in the CG group was 57%.

### Western blot analysis

**Fig 5:**
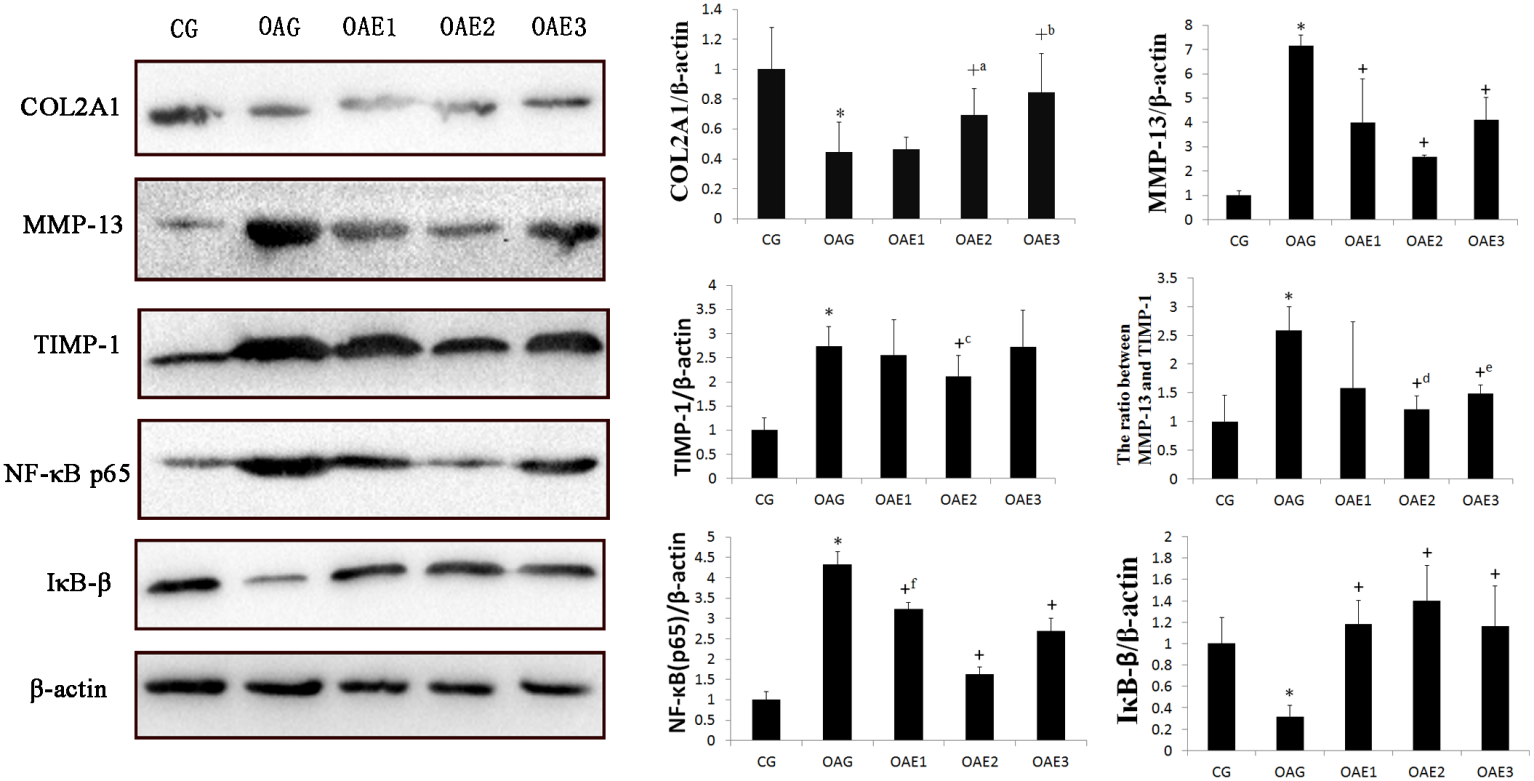
Western blotting results in each group. Effect of different frequency with same amount of treadmill exercise on the expression of MMP-13, TIMP-1, NF-κB p65, IκB-β, and COL2A1 expression in knee joint cartilage of rats with MIA-induced OA. Protein expression was determined in western blots of total protein extracted from cartilage tissues as described in Materials and Methods. The data was obtained in three separate experiments β-actin as an internal standard. Differences between CG and the OAG group were significant (*P < 0.001), and differences between the OAG and the treadmill frequency groups were significant (^+^*P*<0.001, ^+a^*P* = 0.030, ^+b^*P* = 0.001, ^+c^*P* = 0.039, ^+d^*P* = 0.010, ^+e^*P* = 0.038, ^+f^*P* = 0.002,). One-way ANOVA, n = 3 rats for each group, means with 95% confidence interval.

Western blots were evaluated for differences in MMP-13, TIMP-1, NF-κB, IκB-β, and COL2A1 protein expression among the groups of rats with MIA-induced knee osteoarthritis. (Fig 5) Expression of MMP-13, TIMP-1, and NF-κB p65 was higher in the knee joint articular cartilage of rats in the OAG than in the other groups. The expression of IκB-β and COL2A1 was lower than the OAG than in the other groups. Fig 5 showed the effects of each frequency of moderate treadmill exercise on protein expression.

### qPCR assay

**Fig 6:**
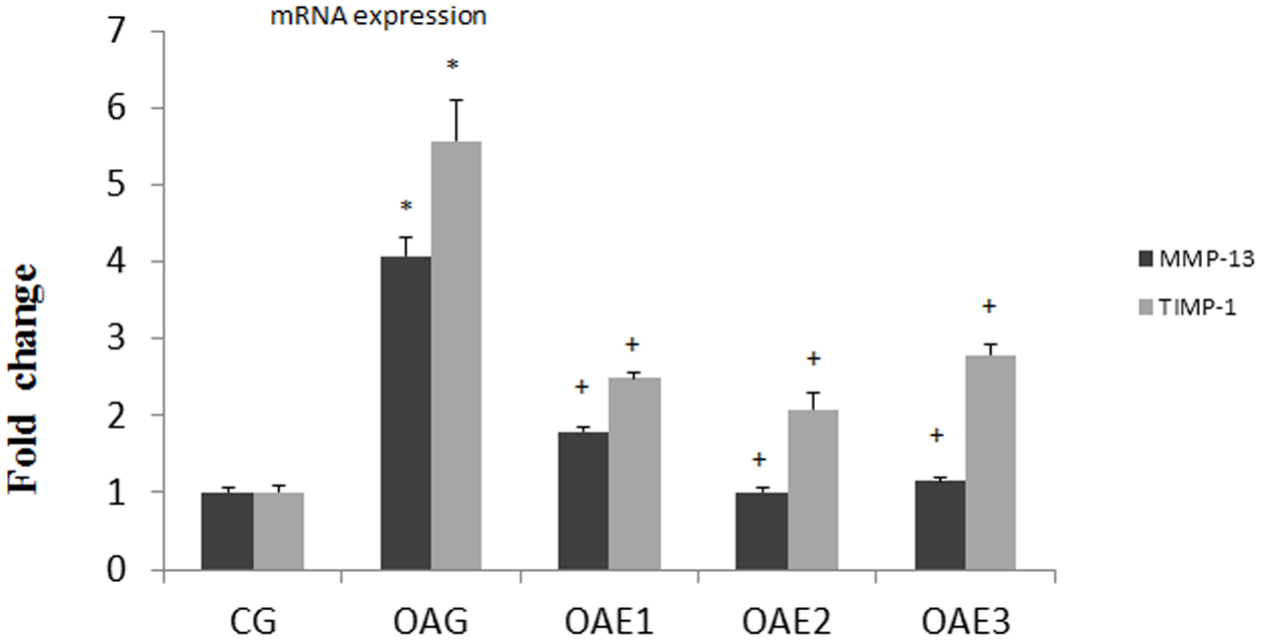
qPCR results in each group. Effect of different frequencies treadmill exercise on the expression MMP-13 and TIMP-1 mRNA in knee joint cartilage from rats with MIA-induced OA. The gene expression of MMP-13 and TIMP-1 was determined by qPCR as described in Materials and Methods. The data were representative of three separate experiments and β-actin was used as an internal standard. Differences between CG and OAG were significant (*P<0.001) and differences between OAG and the different treadmill frequency groups were significant (^+^*P*<0.001). One-way ANOVA, n = 9 rats in each group, means with 95% confidence interval.

Relative expression of MMP-13 and TIMP-1 mRNA is shown in Fig 6. The expression of both TIMP-1 and MMP-13 was higher in the OAG than in the CG group, and expression of TIMP-1 and MMP-13 in the different treadmill frequency groups was lower than that in the OAG (mRNA of MMP-13: CG = 1.00–95% CI 0.98–1.02; OAG = 3.96–95% CI 3.80–4.12; OAE1 = 1.85–95% CI 1.84–1.86; OAE2 = 1.00–95% CI 0.94–1.07; OAE3 = 1.15–95% CI 1.10–1.20. mRNA of TIMP-1: CG = 1.00–95% CI 0.94–1.06; OAG = 5.57–95% CI 5.05–6.09; OAE1 = 2.44–95% CI 2.40–2.49; OAE2 = 2.03–95% CI 1.89–2.17; OAE3 = 2.78–95% CI 2.63–2.93).

## Discussion

In this study, OA was induced by intra-articular injection of MIA, which causes histological changes including cartilage surface erosion, matrix loss and chondrocyte death. In all the OAE groups, OAE3 had better therapeutic effects compared with other OAE groups, which as indicated by histological scores.

Physical activity is one of the most widely applied non-pharmacological therapies for OA. Molecular studies have revealed that specific biomechanical stimuli and cell-cell interactions generate intracellular signals that are powerful inducers or suppressors of pro-inflammatory genes in chondrocytes [38]. Cartilage matrix degradation is mediated primarily by two major families of proteolytic enzymes, MMPs and TIMPs [11,39]. MMP-13 is the most potent enzyme for cleaving type II collagen, the principal form in articular cartilage, whereas TIMP-1 has been shown to inhibit MMPs in rat models [40]. As inflammatory cytokines, IL-1β and TNF-α, contribute to repressing the expression of COL2A1, which is essential for the maintenance, strengthening, and regeneration of cartilage, and to activating MMP-13 transcription in chondrocytes [41]. All frequencies of treadmill exercise were able to decrease the expression of IL-1β, TNF-α, MMP-13, and NF-κB p65. These results corroborate previous findings that moderate treadmill exercise can alleviate the severity of cartilage lesions in experimental OA through its anti-inflammatory capacities [28,30].

Several reports have demonstrated a direct anti-inflammatory effect of moderate treadmill exercise on knee OA *in vivo* [42]. The results showed that moderate treadmill exercise increased the concentrations of LXA_4_ in serum and intra-articular lavage fluid and decreased the concentration of IL-1β, TNF-α, MMP-13, and NF-κB, indicating alleviation of cartilage damage. Because body weight also influenced OA, we measured the body weight of SD rats at regular intervals. The results showed that body weight decrease significant in OAE groups compared to OAG, but there were no significant difference among OAE groups (S2 Fig). Regarding the frequency of exercise, the histopathology findings indicated that OAE3 had a better therapeutic effect than OAE1 and OAE2 on the tibiofemoral joint.

LXA_4_ is a potent stop signal of inflammation [13] and the therapeutic effect of LXA_4_ on OA has been confirmed by previous studies [14–16]. Our preliminary experiment found that LXA_4_ was rapidly elevated in 2 hours and rapidly reduced to normal level at 4 hours after treadmill exercise. And the variation of LXA_4_ was similar after 20 min, 30 min and 60 min treadmill exercise (S1 Fig). The intervals between two treadmill exercises exceeded 4 hours. Different frequencies treadmill exercises had different times to increase the level of LXA_4_. OAE3 could increase the level of LXA_4_ more times than OAE1 and OAE2, so it may be the reason for OAE3 obtained a better result. The overall results suggested that treadmill exercise had therapeutic effects on MIA-induced OA possibly acting via the anti-inflammatory activity of LXA_4_.

**Fig 7:**
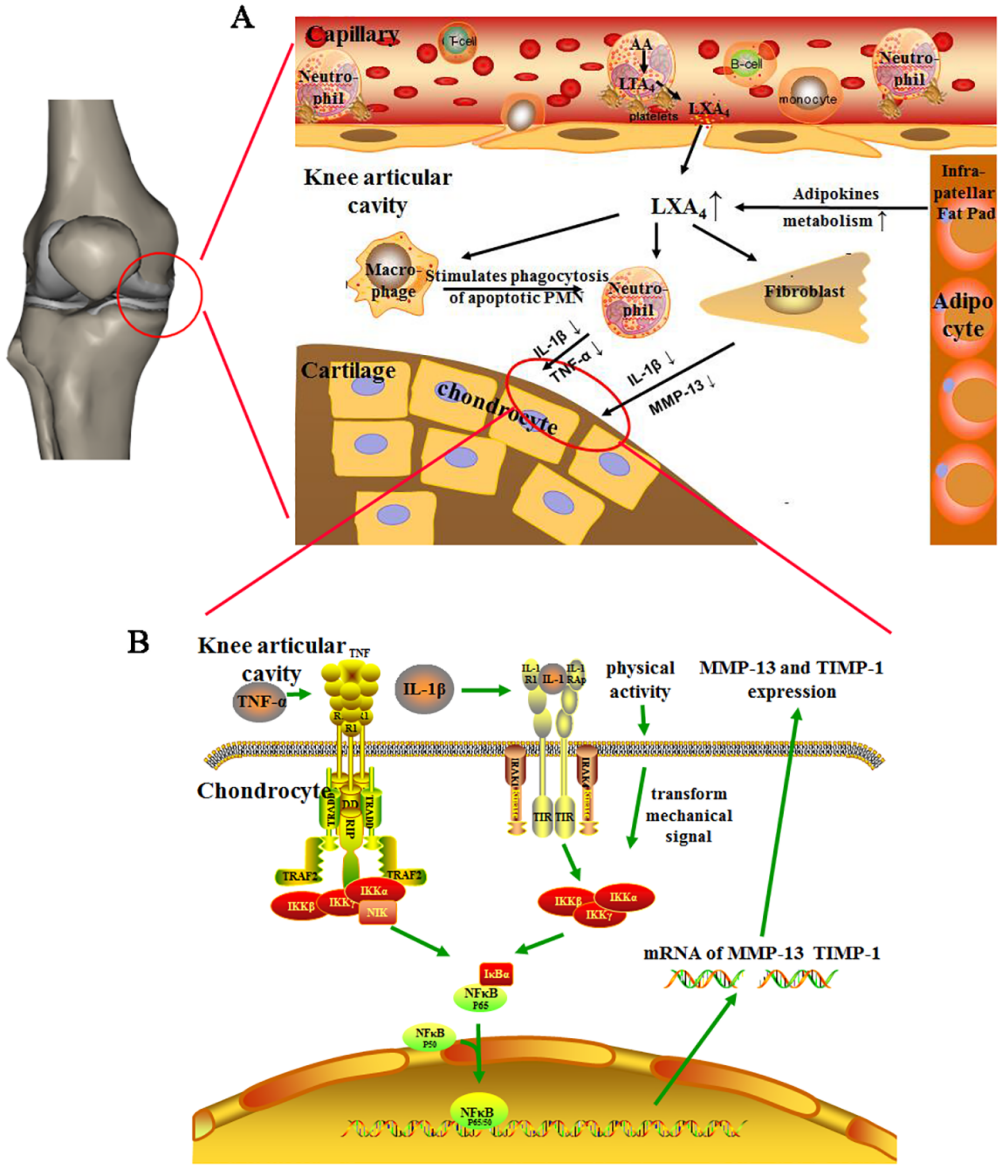
The LXA_4_ and NF-κB pathways play a key role in chondroprotective effect of treadmill exercise. (A) Moderate-intensity exercise promotes platelet/ neutrophils aggregation in capillaries and adipokine metabolism in the infrapatellar fat pad, which upregulate LXA_4_ level in the knee articular cavity. LXA_4_ interaction with macrophages, neutrophils, and synovial fibroblasts lead to the downregulation of IL-1β, TNF-α and MMP-13. (B) Chondrocytes, as mechanosensitive cells, can transform mechanical signals. Thus, exercise decreases the levels of IL-1β and TNF-α via action of LXA_4_, and mechanical signals transduced by chondrocytes inhibit the expression of MMP-13 and TIMP-1 mRNA to protect cartilage via the NF-κB pathway.

Upregulation of LXA_4_ in the knee articular cavity by treadmill exercise might be proceed by promotion of platelet/polymorphonuclear neutrophil aggregation in capillaries, which generates LXA_4_ through the action of 12-lipoxygenase [17]. On the other hand, it might be caused by activation of the oxylipin pathway of the infrapatellar fat pad [18]. LXA_4_ can interact with macrophages, neutrophils and synovial fibroblasts to inhibit the synthesis of inflammatory cytokines, such as IL-1β and TNF-α in the articular cavity. (Fig 7A) It should be mentioned that TIMP-1, a chondroprotective enzyme, also decreased with moderate exercise, which is not consistent with previous reports [40]. However, the ratio of MMP-13 to TIMP-1 decreased, consistent with the observed histological changes, which possibly indicated negative feedback regulation of MMP-13.

NF-κB signaling orchestrates most inflammatory responses and participates in the transcriptional regulation of proinflammatory genes that are stimulated in chondrocytes by mechanical signals [43]. IκB has both cytoplasmic and nuclear roles in regulating the NF-κB pathway [44]. The expression of NF-κB is correlated with that of MMP-13 and is involved in the degradation of the extracellular matrix (ECM) of articular cartilage. Low-to-moderate mechanical stress and LXA_4_ might prevent nuclear translocation of NF-κB, resulting in the inhibition of proinflammatory gene expression [43]. The data showed that a shift to nuclear localization of NF-κB occurred in chondrocytes during cartilage destruction in MIA-induced OA. Activation of IκB-β and inhibition of the expression of NF-κB, resulting in potent inhibition of MMP-13 gene expression could explain the preservation of cartilage and increase COL2A1 content associated with treadmill exercise. (Fig 7B)

In conclusion, under the condition of the 60 min treadmill exercise with moderate-intensity, to fulfill in three times would have better chondroprotective effects than to fulfill in two or one time on MIA-induced OA in rats. And it maybe worked through the anti-inflammatory activity of LXA_4_ and the NF-κB pathway. The results provide new insight to formulate improved exercise programs for further study of the optimum exercise and LXA_4_ maybe is a significant indicator to detect the effect of treadmill exercise.

## Supporting information

S1 Fig Impact of different duration treadmill exercise on the levels of LXA_4_ of serum and intra-articular lavage fluid. Fifty-two SD rats were divided into four groups. The basal group was no treadmill exercise (n = 4). The rest of SD rats were divided into 20min, 30min and 60min treadmill exercise group respectively with moderate-intensity (speed: 18m/min, n = 16). Serum and intra-articular lavage fluid were collected immediately after the exercise (A.E.), and also 1, 2, 4 h after exercise. Differences between the basal and 20min treadmill exercise (^+^*P* <0.001, ^+a^*P* = 0.040), 30min treadmill groups (*P<0.001, *^a^*P* = 0.038, *^b^*P* = 0.006, *^c^*P* = 0.038, ^+e^*P* = 0.044), and 60min treadmill groups (^#^*P*<0.001, ^#a^*P* = 0.002, ^#b^*P* = 0.005) were significant. But there were no significant different at A.E., 1h, 2h and 4h among 20min, 30min and 60min treadmill exercise in serum and intra-articular lavage fluid. One-way ANOVA, n = 4 rats for each group, means with 95% confidence interval.
(TIF)

S2 Fig Effect of different frequency with same amount of treadmill exercise on body weight. SD rats were divided into five groups (n = 10 per group). Rats in all groups received a standard diet. Body weights were measured weekly for 10 weeks. Differences between CG and OAE1 were significant (^+^*P* <0.001, ^+a^*P* = 0.003), differences between CG and OAE2 were significant (*P<0.001, *^a^*P* = 0.003), and differences between CG and OAE3 were significant (^#^*P*<0.001, ^#a^*P* = 0.001, ^#b^*P* = 0.007, ^#c^*P* = 0.001). But there were no significant among OAE groups. One-way ANOVA, n = 10 rats for each group, means with 95% confidence interval.
(TIF)
