# Supplementary figures and images for: The effects of different frequency treadmill exercise on lipoxin A4 and articular cartilage degeneration in an experimental model of monosodium iodoacetate-induced osteoarthritis in rats
Source: PLoS One. 2017 Jun 8;12(6):e0179162. doi: 10.1371/journal.pone.0179162 (PMC5464632; doi:10.1371/journal.pone.0179162)

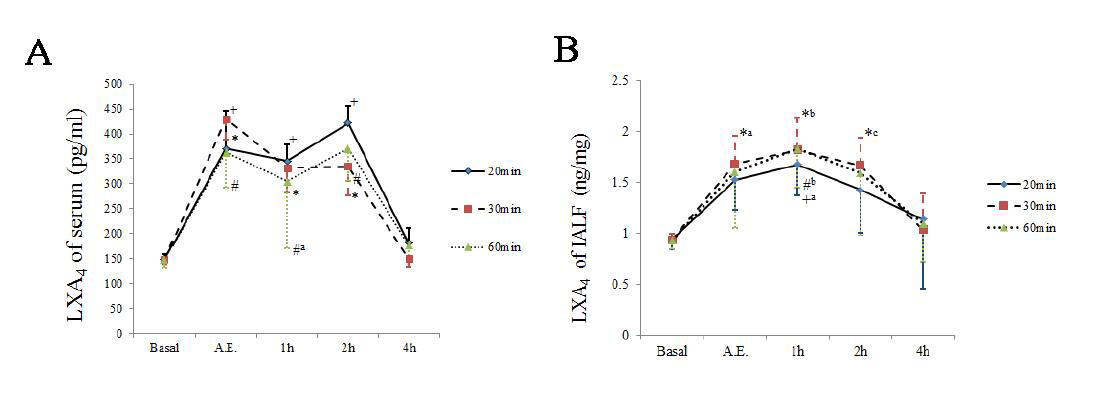

Supplement: S1 Fig — Fifty-two SD rats were divided into four groups. The basal group was no treadmill exercise (n = 4). The rest of SD rats were divided into 20min, 30min and 60min treadmill exercise group respectively with moderate-intensity (speed: 18m/min, n = 16). Serum and intra-articular lavage fluid were collected immediately after the exercise (A.E.), and also 1, 2, 4 h after exercise. Differences between the basal and 20min treadmill exercise (+P <0.001, +aP = 0.040), 30min treadmill groups (*P<0.001, *aP = 0.038, *bP = 0.006, *cP = 0.038, +eP = 0.044), and 60min treadmill groups (#P<0.001, #aP = 0.002, #bP = 0.005) were significant. But there were no significant different at A.E., 1h, 2h and 4h among 20min, 30min and 60min treadmill exercise in serum and intra-articular lavage fluid. One-way ANOVA, n = 4 rats for each group, means with 95% confidence interval. (TIF) [file pone.0179162.s001.tif]

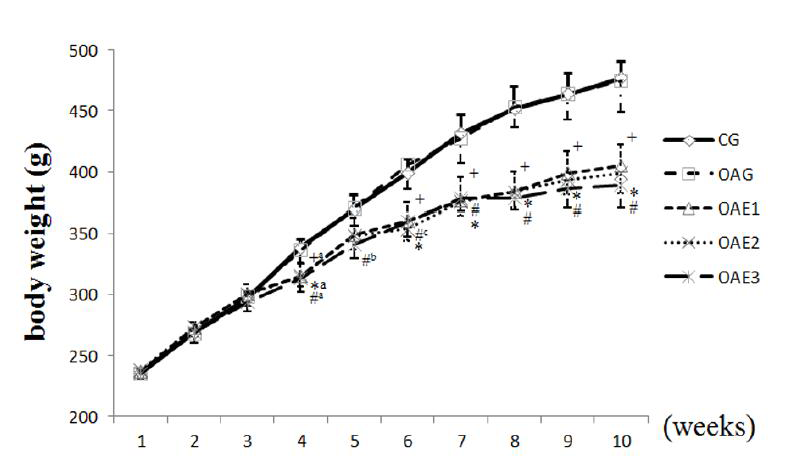

Supplement: S2 Fig — SD rats were divided into five groups (n = 10 per group). Rats in all groups received a standard diet. Body weights were measured weekly for 10 weeks. Differences between CG and OAE1 were significant (+P <0.001, +aP = 0.003), differences between CG and OAE2 were significant (*P<0.001, *aP = 0.003), and differences between CG and OAE3 were significant (#P<0.001, #aP = 0.001, #bP = 0.007, #cP = 0.001). But there were no significant among OAE groups. One-way ANOVA, n = 10 rats for each group, means with 95% confidence interval. (TIF) [file pone.0179162.s002.tif]
